# Patterns of Warfarin Use in Subgroups of Patients with Atrial Fibrillation: A Cross-Sectional Analysis of 430 General Practices in the United Kingdom

**DOI:** 10.1371/journal.pone.0061979

**Published:** 2013-05-02

**Authors:** Mohammed A. Mohammed, Tom Marshall, Krishnarajah Nirantharakumar, Andrew Stevens, David Fitzmaurice

**Affiliations:** School of Health and Population Sciences, University of Birmingham, Edgbaston, Birmingham, United Kingdom; Cardiff University, United Kingdom

## Abstract

**Background:**

Despite the proven efficacy of warfarin, its use in patients with Atrial Fibrillation (AF) is reportedly low. We investigated the underuse and overuse of warfarin in the management of AF in general practices in the United Kingdom (UK) against the National Institute of Clinical Excellence (NICE, UK) guidelines whilst seeking to identify subgroups of AF patients to inform efforts to optimise warfarin use.

**Methodology:**

A retrospective database analysis to determine warfarin prescribing using tree models based on 50361 patients with AF (classified as low, moderate and high risk of stroke using CHADS2) from 430 general practices in the UK.

**Results:**

Over one-third (37.0%, 4573/12351) of low risk AF patients were on warfarin, compared with 47.1% (8349/17709) moderate risk AF patients and 54.9% (11142/20301) high risk AF patients. Clinical subgroups (n = 15 low risk subgroups, n = 15 medium risk subgroups, n = 22 high risk subgroups) were identified. Several factors not supported by current guidelines (age, BMI, dementia, gender) were associated with the use of warfarin. Gender and BMI were associated with warfarin use in low and medium risk AF patients but not in high risk AF patients.

**Conclusion:**

Whilst NICE guidelines suggest that all high risk AF patients should be on warfarin, half of those at moderate risk should be on warfarin and none of those at low risk should be on warfarin, we found evidence of over and under use of warfarin. Interventions to optimise warfarin therapy tailored to and targeting specific subgroups of AF patients identified by the tree models are required.

## Introduction

Atrial Fibrillation (AF) is the commonest sustained arrhythmia encountered in clinical practice [1], [2]. The prevalence of AF increases with age and is higher in men than women [2], [3]. It may be occasional and self-limiting (paroxysmal), persistent or permanent [2], [4]. AF increases mortality and hospitalisation rates [2], [5], [6], [7], [8] and is also associated with a five-fold increase in the risk of stroke [2], [9], [10].

Treatment with aspirin reduces the risk of stroke by about one quarter and anticoagulant treatment with adjusted dose warfarin reduces this risk by about two thirds [2], [11], [12], [13]. So whilst the efficacy of warfarin is established, warfarin is reportedly underused, perhaps because of concern that the risk of bleeding is higher with warfarin than aspirin [2], [14]. But evidence suggests that the benefits of warfarin outweigh its harms in most patients, including the elderly [2], [5], [15].

Guidelines stratify patients with AF according to their risk of stroke in order to support treatment decisions [2], [8], [16], [17], [18]. The most widely used stroke risk stratification tool is the CHADS2 score [19]. Anticoagulants are recommended for those at high risk (CHADS2 score >1), anticoagulants or aspirin for medium risk (CHADS2 score 1) and aspirin for low risk (CHADS2 score 0). Although recent guidelines include newer anticoagulants and a modified scoring system (CHA_2_DS_2_-VASc), the key recommendations concerning anticoagulation remain essentially unchanged [20], [21].

Clinical guidelines from the National Institute for Clinical Excellence (NICE), presently under review, suggest that all high risk AF patients should be on warfarin, half of those at moderate risk should be on warfarin and none of those at low risk should be on warfarin [22]. Studies from several different countries have found that substantial proportions of patients eligible for warfarin are not prescribed warfarin [23]. High rates of warfarin use have been reported from Germany and Switzerland [24], [25]. However some of these studies have been small or confined to a few health care providers. They tend to focus on underuse of warfarin and have not considered the clusters of patient characteristics that are associated with underuse or overuse of warfarin. Identifying clinical subgroups of patients more likely to be under or over treated is useful in understanding the clinical reasoning which may have led to treatment decisions. This may be useful in understanding suboptimal clinical decision making, informing targeted improvement strategies or identifying gaps in the underlying evidence base (e.g. the evidence of efficacy in patients with particular characteristics).

Using the CHADS2 scoring system (and, as a sensitivity analysis, the CHA_2_DS_2_-VASc scoring system) to identify AF patients eligible and ineligible for anticoagulants we analysed warfarin anticoagulant prescribing patterns across 430 general practices in the United Kingdom (UK). Our aim was to determine adherence to clinical guidelines and to identify the characteristics of subgroups of AF patients associated with the overuse and underuse of anticoagulants.

## Methods

### Ethics approval

Ethical approval (reference 08/H0305/3) was obtained by TM from the National Research Ethics Service for the National Health Service (NHS).

### Data source

Our data originate from The Health Improvement Network (THIN), an electronic database of medical records uploaded from general practices using the VISION computer system. There are currently 2.2 million active patients in over 400 practices: 4.7 million patients when historical data are included. The THIN database is subject to frequent internal quality checks, with any practices failing to maintain adequate quality standards removed from the database. The records contain patient characteristics, all prescriptions, consultations, diagnoses and primary care investigations.

Only patients with a diagnosis of AF were selected based on the occurrence of appropriate Read Codes (available via the corresponding author). Patients aged less than 35 years of age or patients with valvular heart disease were excluded because anticoagulants are rarely indicated in the former and always in the latter. All patients in the database on 1st May 2010 were included in the analysis provided they had least one year of records prior to the index date and the practice records fell within a period of acceptable mortality data quality recording. [26]

CHADS2 scores were calculated for all AF patients: one point was added for a history of heart failure, hypertension, age ≥75 years and diabetes and two points for a history of stroke or transient ischaemic attack or thromboembolic event. Hypertension was defined as either a current prescription for antihypertensive drugs or the mean of the three most recent systolic blood pressures in the past three years ≥160 mm Hg. Patients were stratified by CHADS2 score: 0, 1 and >1. The CHADS2 score was not in use before 2009 however the scoring system was used to stratify patients according to their risk of stroke in order to provide insights into the anticoagulant therapy. Considering the CHA_2_DS_2_-VASC was introduced into clinical practice by the European Society of Cardiology [21] in the latter part of 2010, we undertook a sensitivity analysis to see if warfarin use would materially change under this scoring system. For CHA_2_DS_2_-VASC score we allocated one point for heart failure, hypertension, diabetes mellitus, age 65 to 74 years, vascular disease (defined as previous MI or peripheral vascular disease), female gender if aged≥65 years and 2 points for stroke, TIA, thromboembolic event and age ≥75 years.

### Definition of variables

Patients were classified as receiving an anticoagulant drug if a prescription for a drug in British National Formulary category 020802 had been issued within 90 days of the index date. Patients were classified as receiving an antiplatelet drug if a prescription for a drug in British National Formulary category 0209 had been issued within 90 days of the index date or there was a record that the patient was taking over the counter aspirin within the past year. [27]

We identified the following candidate set of patient level covariates which might influence the anticoagulation prescribing, many of which were identified in a recent narrative [2] and systematic review [28]:- patients age (years), gender (male/female) and the presence/absence of any of the following: aspirin use, current smoker, excessive alcohol use, diabetes, bleeding history or bleeding disorders, ischaemic heart disease, peptic ulcer disease, renal disease, hyperthyroidism, hypothyroidism, gastro-oesophageal reflux, gastritis, family history of coronary heart disease, body mass index (BMI Kg/m^2^), hypertension, heart failure, stroke, transient ischaemic attack, angina, myocardial infarction, peripheral vascular disease, history or at high risk of falls, dementia and liver disease. Signs, symptoms and diagnosis terms were defined using the Read Codes.

### Statistical analysis using tree models

**Figure 1:**
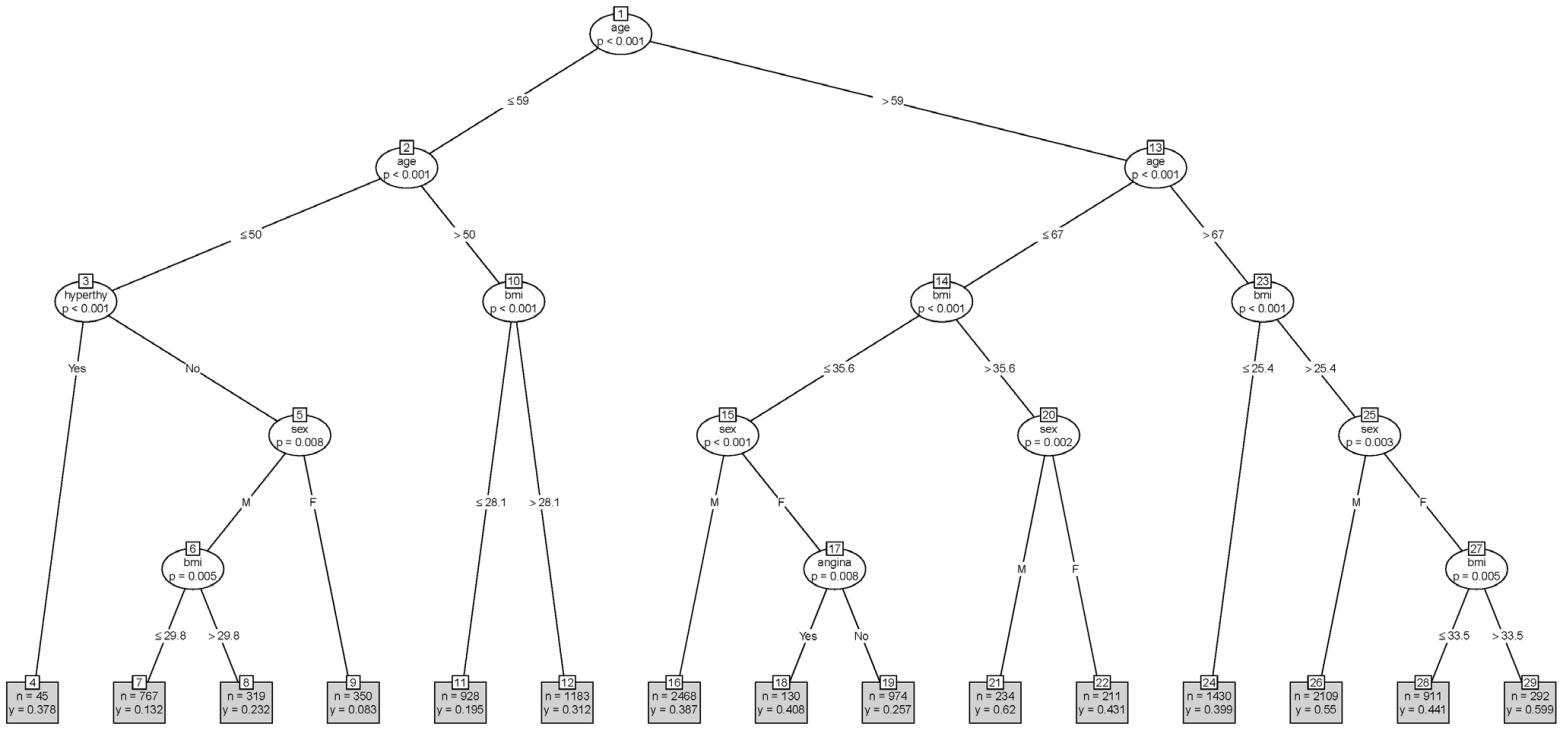
Warfarin prescribing tree for low risk AF patients. Hyperthy is hyperthyroidism. Bmi is body mass index.

**Figure 2:**
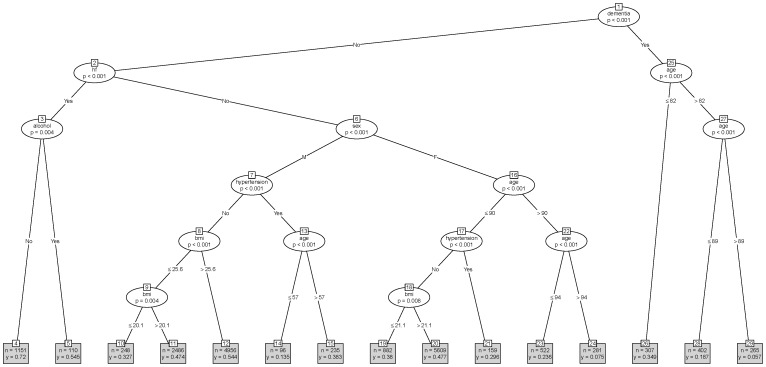
Warfarin prescribing tree for medium risk AF patients. ihd is ischemic heart disease. Bmi is body mass index. hf is heart failure.

**Figure 3:**
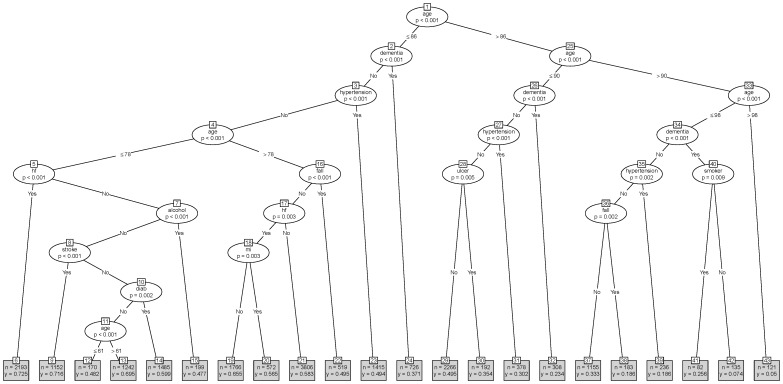
Warfarin prescribing tree for high risk AF patients. hf is heart failure. mi is myocardial infarction. tia is transient ischemic attack. diab is diabetes.

Our primary analysis involves the use of Classification and Regression Trees, (CART), which are a statistical data mining knowledge discovery technique which produce trees "explaining" AF prescribing patterns based on a set of covariates by recursively splitting or partitioning patients into homogenous groups [29]. Although their use is still somewhat novel they have been used to support medical decision making [30], [31], [32] especially because they may identify clinically meaningful subgroups. Our use of tree models here is exploratory, intended to generate hypotheses about complex AF prescribing decisions based on a candidate set of covariates. Tree models can deal with nonlinear relationships, high-order interaction effects and missing values, whist producing simple to understand results that are distribution free. Trees explain variation of a single response variable (AF Prescribing yes/no in our case) by repeatedly splitting the data into more homogeneous groups using combinations of candidate variables. When first developed, CARTs, could lead to quite large tree models, but recent work has incorporated p-value based tree modelling, known as conditional trees, which yield smaller tree models whilst simultaneously controlling for multiple testing, (Bonferroni adjustment, based on p≤0.01). They are available in the *Party* Package [33] in *R* [34]. Our purpose in using conditional tree models is to identify subgroups of AF patients stratified by CHADS2 risk (low (figure 1), medium (figure 2), high (figure 3) without seeking to develop a clinical prediction model [30]. We also produced tree models stratified by the CHA2DS2-VASC risk (see figures S1, S2, and S3 which show tree models for warfarin prescribing when AF patients are classified into low, medium and high risk using the newer CHA_2_DS_2_-VASc risk score respectively). In navigating a tree model we use the node numbers to identify pathways leading to terminal nodes which contain clinical subgroups with sample sizes (n) and proportions of AF patients on warfarin (y).

## Results

There were 50361 patients with AF from 430 general practices. The mean age of these patients was 75.6 years (standard deviation (SD) 11.7) and over half (55.9%, 28153/50361) were male. About one quarter of these patients were low risk (24.5% 12351/50361) for stroke (according to the CHADS2 score) whilst 35.1% (17709/50361) were moderate risk and 40.3% (20301/50361) were high risk. Table 1 shows warfarin use under the CHADS2 risk score and the CHA_2_DS_2_-VASc risk score and Table 2 provides a breakdown by gender and age <75 years.

**Table 1:** Warfarin use (%) in AF patients stratified by CHADS2 and CHA2DS2-VASc risk groups.

	Warfarin		
CHADS2 Risk Category	No (%)	Yes (%)	Total
Low Risk	7778 (62.97)	4573 (37.03)	12351
Medium Risk	9360 (52.85)	8349 (47.15)	17709
High Risk	9159 (45.12)	11142 (54.88)	20301
**CHA_2_DS_2_-VASc Risk Category**			
Low Risk	4126 (73.44)	1492 (26.56)	5618
Medium Risk	2787 (53.07)	2465 (46.93)	5252
High Risk	19384 (49.08)	20107 (50.92)	39491

**Table 2:** Warfarin use (%) in AF patients stratified by CHADS2 and CHA2DS2-VASc risk groups and gender and age<75 years.

	Males <75 years	Males ≥75 years	Females <75 years	Females ≥75 years
**CHADS2 Risk Category**	N = 13806	N = 14347	N = 6606	N = 15602
Low Risk	3222/8281 (38.91)	-	1351/4070 (33.19)	-
Medium Risk	1804/3205 (56.29)	3069/6167 (49.76)	641/1330 (48.20)	2835/7007 (40.46)
High Risk	1600/2320 (68.97)	4649/8180 (56.83)	806/1206 (66.83)	4087/8595 (47.55)
**CHA_2_DS_2_-VASc Risk Category**				
Low Risk	1166/3998 (29.16)	-	326/1620 (20.12)	-
Medium Risk	2333/4866 (47.94)	-	132/386 (34.20)	-
High Risk	3127/4942 (63.27)	7718/14347 (53.8)	2340/4600 (50.87)	6922/15602 (44.37)

Blank cells reflect a not applicable cell.

Table 1 shows that warfarin use ranged from 37.03% to 54.88% under the CHADS2 risk groups and was relatively low (26.56% to 50.92%) under the CHA2DS2-VASC risk score. Table 2 further stratifies the use of warfarin by gender and age <75 years, which showed that females had lower warfarin use than males in all risk groups.

Of those AF patients not on warfarin, 43.1% (3354/7778) in the low risk CHADS2 category were not on aspirin either although this declined to 28.4% (2659/9360) in the medium risk CHADS2 category and 22.3% (2043/9159) in the highest risk CHADS2 category. Under the CHA_2_DS_2_-VASc score these figures were 53.9% (2227/4126, low risk), 34.4% (958/2787, medium risk) and 25.1% (4871/19384, high risk) respectively.

### Low risk AF patients

In the 12351 low risk AF patients, 37.03% (4573/12351) were on warfarin and of those not on warfarin, over half (56.88%, 4424/7778) were on aspirin. Under the CHA_2_DS_2_-VASc score the figures are 26.56% (1492/5618) and 46.03% (1899/4126) respectively.

**Figure 4:**
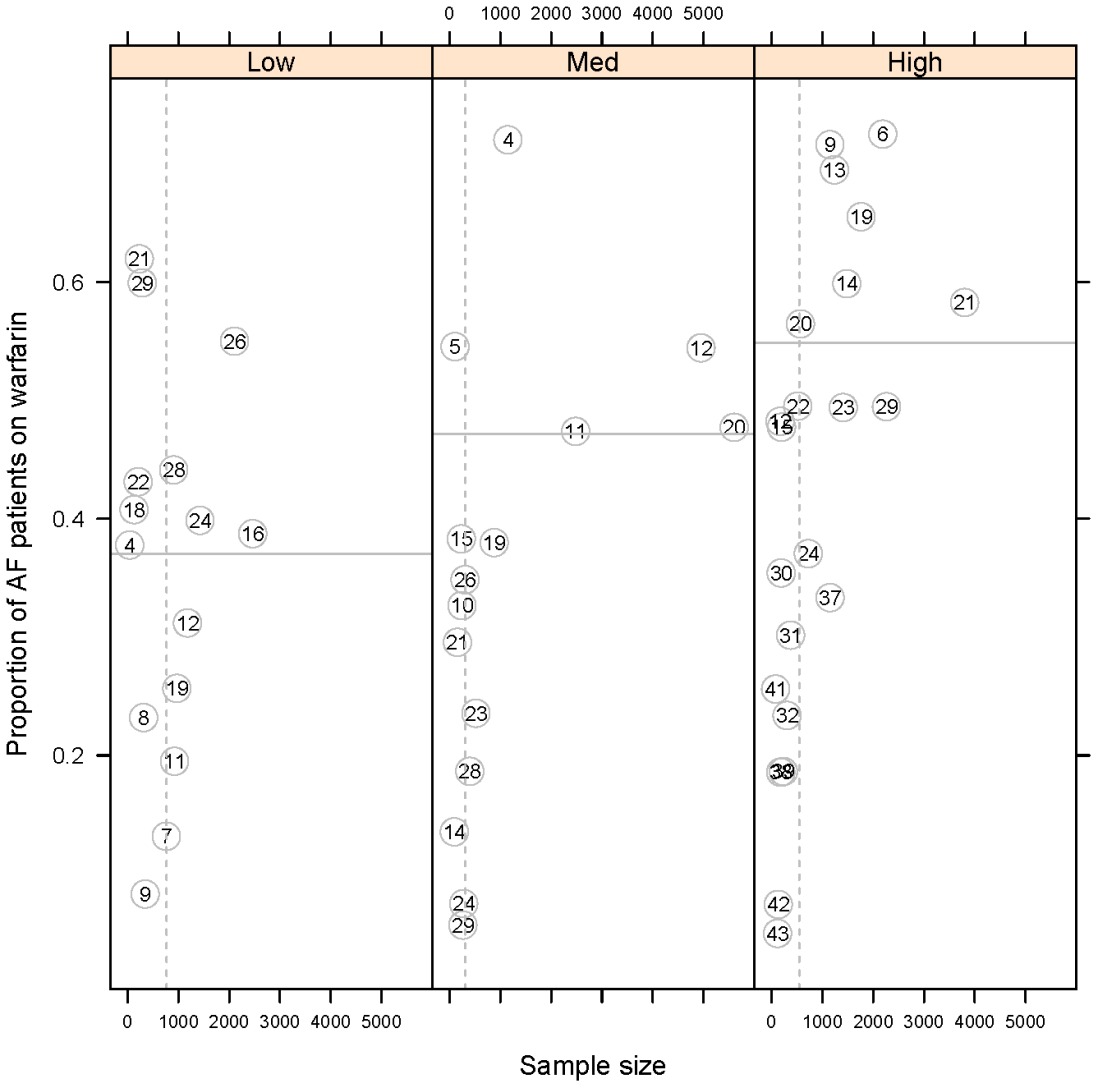
Scatter plots showing proportion of AF patients on warfarin and sample size in all the terminal nodes from the tree models by CHADS2 risk groups. Terminal node numbers shown in circles. Horizontal line is the average proportion. Vertical dotted line is the median sample size.

For low risk AF patients the tree model (see figure 1) identified the following patient variables as being significant predictors of warfarin use: age, hyperthyroidism, BMI, gender and angina culminating in 15 clinical subgroups (located in the 15 terminal nodes). Besides hyperthyroidism (node 3) and angina (node 17) all other branches in the tree model contained combinations of age, sex and BMI demonstrating significant interaction effects and non-linear relationships with warfarin use. Figure 4 (left panel) shows the information (proportion on warfarin and subgroup sample size) contained in the 15 terminal nodes in a scatter plot (to aid visualisation). The primary node in this tree split on age ≤59 years and shows how clinical practice appears to vary with this age split.

In patients aged >59 years (node 1), the use of warfarin ranged from 25.7% (node 19) to 59.9% (node 29). Node 19 contained females aged between 59 and 67 years with BMI≤35.6 and no angina; whereas node 29 contained females aged >67 years with BMI >33.5. Low risk AF patients located in terminal nodes 21, 29 and 26 had the highest warfarin use (62%, 59.9%, 55% respectively). These patients were over 59 years of age, had high BMI values which interacted with the patients gender. Node 26 contained one of the largest subgroups of low risk patients (n = 2109) associated with 55% warfarin use. This subgroup of patients was aged >67 years, overweight (BMI>25.4) and male. In some instances (node 21 vs node 22) males had higher warfarin use and in other instances females had higher use (node 29 vs 26, node 18 vs node 16). Females patients in general had lower warfarin use than males (nodes 5, 15) except if they had angina (node 17), where female patients with angina (node 18 vs node 19) also had significantly higher warfarin use than female patients without angina (40.8% vs 25.7%). Higher BMI values were associated with significantly higher warfarin use (node 27, node 23 and node 14).

In patients age ≤59 years (node 1) the use of warfarin ranged from 8.3% (node 9) to 37.8% (node 4), although the latter contained only 45 low risk AF patients. Node 9 had females aged ≤50 years and node 4 had AF patients with hyperthyroidism aged ≤50 years. Higher BMI values were associated with significantly higher warfarin use (node 10 and node 6).

### Medium risk AF patients

In the 17709 medium risk AF patients 47.15% (8349/17709) were on warfarin and of those not on warfarin, 71.59% (6701/9360) were on aspirin. Under the CHA_2_DS_2_-VASc scoring system the figures are 46.93% (2465/5252) and 65.63% (1829/2787) respectively.

For medium risk AF patients, the tree model (figure 2) identified 29 nodes, with 15 clinical subgroups located in the 15 terminal nodes. This tree model identified dementia as the primary node and subsequently included the following variables–heart failure, age, alcoholism, sex, hypertension and BMI–usually in more than one branch of the tree indicating interaction effects and in the case of age and BMI also indicating non-linear relationships with warfarin use. Figure 4 (middle panel) shows the information contained in the terminal nodes in a scatter plot (to aid visualisation). The primary split of this tree is on dementia and shows how clinical practice appears to vary by dementia.

Patients with dementia (node 1) aged ≤82 years had the highest use of warfarin (node 26, 34.9% warfarin use) whilst patients with dementia aged >89 had the lowest use (node 29, 5.7% warfarin use). In patients without dementia, warfarin use ranged from 7.5% (node 24) to 72% (node 4). Node 4 contained patients with heart failure but no dementia and no alcoholism. Node 24 contained female patients aged >94 years. Over half (54.5%) of medium risk AF patients with heart failure and alcoholism were on warfarin (node 5). AF patients with higher BMI values were more likely to be on warfarin than those with lower BMI values (eg node 9 and node 18). The largest subgroups of AF patients were in node 20 (n = 5609, 47.5% warfarin use) and node 12 (n = 4956, 54.4% warfarin use). Node 20 identified female patients with BMI >21.1, aged <90 years with no dementia and no hypertension. Node 12 identified males with BMI >25.6, with no dementia, no heart failure and no hypertension. In general, higher BMI values were associated with increased warfarin use although in a non-linear fashion. Node 6 of the tree splits on gender and subsequent nodes identify age, hypertension and BMI as significant predictors for males and females but in different ways. For non-hypertensive males (node 7) and females (node 18) as BMI increased so too did warfarin prescribing, but different age-cut offs were seen for females (node 16, age 90 years, node 22 age 94 years) compared to males (node 13, age 57 years).

### High risk AF patients

In the 20301 high risk AF patients, 54.88% (11142/20301) were on warfarin on warfarin and of those not on warfarin, 77.7% (7116/9159) were on aspirin. Risk stratification based on CHA_2_DS_2_-VASc score made little difference to this finding (50.92%, 20107/39491).

For high risk AF patients, the tree model (figure 3) identified 22 clinical subgroups reflected in the 22 terminal nodes. This tree model identified age >86 years as the primary node and subsequently included the following variables–age, dementia, hypertension, heart failure, history of falls, ulcer, current smoker, alcoholism, stroke, MI and diabetes but not BMI and gender (unlike the previous trees). Figure 4 (right panel) shows the information (proportion on warfarin, sample size) contained in the terminal nodes in a scatter plot (to aid visualisation). The primary split of this tree is on age ≤86 years and shows how clinical practice appears to vary by this age split.

Nodes to the left of the primary node are AF patients aged ≤86 years. Here patients with dementia (node 24, 37.1% on warfarin), hypertension (node 23, 49.4% on warfarin), alcoholism (node 15, 47.7% on warfarin), history of falls (node 22, 49.5% on warfarin) and those aged ≤61 years (node 12, 48.2% on warfarin) have less than 50% warfarin use. The highest warfarin use is seen in AF patients aged ≤78 years with heart failure (node 6, 72.5% on warfarin) or stroke (node 9, 71.6% on warfarin).

Nodes to the right of the primary node are AF patients aged >86 years. Here the lowest warfarin use was seen in patients aged >98 years (node 43, 5% on warfarin) and patients with dementia aged ≤98 years (node 42, 7.4% on warfarin). Interestingly patients with dementia who smoked were more likely to be on warfarin than those who did not smoke (node 41 vs 42, 25.6% vs 7.4%). The highest use of warfarin was seen in patients aged between 86 and 90 years who had no dementia, no hypertension and no peptic ulcer disease (node 29, 49.5% warfarin use). Patients aged between 86 and 90 years with a history of falls but no dementia and no hypertension were less likely to be on warfarin (node 37 vs node 38, 33.3% vs 18.6% on warfarin).

The two largest subgroups in this tree model (figure 3) are located in terminal node 21 (n = 3806) and 29 (n = 2266) with 58.3% and 49.5% on warfarin respectively. These subgroups are characterised by the absence of any other risk factors in the tree (no dementia, no hypertension, no heart failure and no peptic ulcer disease).

## Discussion

NICE guidelines suggest that all high risk AF patients should be on warfarin, benefits of treatment also clearly outweigh risks in moderate risk and more recent guidelines recommend they are also prescribed warfarin and those at low risk should not be on warfarin. Our analysis showed clear evidence of over prescribing (37% prescribed in low risk group) and under prescribing (45% not prescribed in high risk group) which is inconsistent with NICE guidelines and ESC guidelines. For moderate risk AF patient's warfarin use (47%) appears to be consistent with NICE guidelines, but as with the other strata, the tree models identified considerable heterogeneity within subgroups of patients (range 5.7% to 72%). These overall findings were not materially different under the newer CHA_2_DS_2_-VASc scoring system adopted by the European Society of Cardiology which recommends anticoagulant use in all moderate and high risk patients based on the newer risk score [2], [35]. Under either the CHADS2 or CHA_2_DS_2_-VASc scoring system a substantial proportion of high risk and moderate risk patients were prescribed neither warfarin nor aspirin.

Our findings, which are consistent with several previous reports [23], [36] underscore the need to optimise AF treatment, although few studies have characterised the clusters of clinical characteristics identifying of patients likely to be undertreated or overtreated. Low risk AF patients were collated into 15 clinical subgroups characterised by the interplay of three factors-age, gender and BMI, with higher BMI values resulting in increased warfarin use. Medium risk AF patients were also collated into 15 clinical subgroups with dementia as the primary node and again with higher BMI resulting in increased warfarin use. The high risk AF patients were collated into 22 clinical subgroups with age >86 years as the primary node. Interestingly the effects of gender and BMI were seen in the low and medium risk AF patients but not the high risk AF patients. It is possible that high BMI and male gender are being perceived as risk factors for thromboembolic stroke and therefore are associated with warfarin prescribing.

There are two potential uses of the tree models reported here. (1) They identify clinical subgroups where efforts to optimise warfarin use may be targeted. For example, interventions to optimise the use of warfarin could focus on the high volume clinical subgroups because they appear, from a public health perspective, to offer the largest gains. Nonetheless it is also important to note that the smaller (as well as larger) nodes can also be used to review the clinical decision making in individual patients. (2) They identify risk factors in the context of other patient characteristics (eg BMI and gender) which are influencing clinical decision making although they are not part of present clinical guidelines. This empirical evidence suggests either a gap in the evidence base or clinical practice which is inconsistent with the guidelines. The former should inform future research priorities (evidence is needed to confirm or refute that these characteristics should influence clinical decision making) whilst the latter should inform efforts to improve adherence to guidelines.

Several strategies to improve adherence to clinical guidelines have been proposed including a clinical decision support software system (known as The Auricle) where GPs, cardiologists and patients to work together to arrive at the optimum warfarin therapy decision. Whilst there is anecdotal evidence that Auricle is useful it is being the subject of a trial 20 general practices and its results are awaited [14]. The clinical subgroups identified here could be targeted by such decision support systems.

NICE estimated that 355000 AF patients were at high risk of stroke in the UK and that 166000 were on warfarin (47%) and it has been argued that if GPs can optimise warfarin use to appropriate AF patients then there is the potential to save at least 12500 strokes per year [22]. But AF therapy is a complex process and optimisation has to address several possible constraints including the possible lack of knowledge among GPs and patients, a disproportionate concern over the side-effects of warfarin (eg bleeds) when compared with the risk of stroke, the use of aspirin (instead of warfarin), and time constraints both to the patient and physician due to the need for long term monitoring [37]. Furthermore the performance incentives for GPs in the National Health Service (NHS), known as Quality Outcomes Framework (QOF), does not discriminate between the use of aspirin and warfarin in AF and this may inadvertently undermine warfarin prescribing, although this is being remedied in the next edition.

In most nodes males were preferentially treated with warfarin when evidence clearly identifies females to be at higher risk of thromboembolic event [2], [35]. The reasons for our finding that patients with higher BMI were more likely to be on warfarin are not clear, even though obese individuals are at higher risk of venous thromboembolic event [38], this is not the case with AF where arterial thromboembolism is the main concern [2]. The higher use of warfarin in AF patients with heart failure is interesting because it may reflect better clinical decision making in this subgroup because their care is shared between primary and secondary care specialists and to some extent parallels the approach in Auricle [14]. Literature suggests hyperthyroid AF patients have similar risks of thromboembolism to that of patients without hyperthyroid diseases and reviews suggest warfarin use in this group of patients should be based on additional risk factors for thromboembolic event [39].

### Limitations

There are several limitations to our study. The clinical subgroups have been identified via a data mining technique and so may not always be clinically meaningful but because they are empirically derived they may still remain informative to efforts to optimise warfarin use. Our study involves a cross-sectional analysis of the THIN database and, like previous studies, does not therefore consider the longitudinal dimension in that the order of events is not known. This bias could be addressed by a retrospective database cohort study design but the evidence of over and under use of warfarin is so overwhelming that such a study appears somewhat unlikely to materially modify the findings. Although we focused on patient characteristics, factors such a patient preference, patient adherence, patient family and social circumstances and severity of disease including risk of bleeding [40] (as opposed to bleeding history or bleeding disorders, which we did consider) were not incorporated because they are generally not well recorded in routinely collected databases. Nonetheless future studies that focus on over/under use of warfarin should endeavour to consider these factors. Whilst the omission of these factors is likely to overestimate the underuse of warfarin, these factors are unlikely to make a material difference to our general findings. We excluded general practice from the tree models as the aim here was to determine current practice against clinical guidelines and the latter make no allowance for general practice, although from a modelling perspective we have overlooked a possible source of variance. Since warfarin therapy has few contra-indications (which we did consider) further studies could also consider patient consent/compliance and poly-pharmacy. Our tree models were based on the CHADS2 scoring system which is relevant to the time period of our data, although in due course, future studies will need to reflect the use of the newer, CHA2DS2-VASc scoring as it gets embedded into routine clinical practice. Nevertheless we have produced tree models under the CHA2DS2-VASc scoring in a supplementary file (Figure S1, Figure S2, and Figure S3). Whilst the extent of warfarin over and under use remained broadly comparable under CHADS2 and CHA2DS2-VASc, almost twice as many AF patients were categorised as high risk under the newer score and this suggests that the underuse of warfarin in high risk patients should be given a high priority.

## Supporting Information

Figure S1 **Warfarin prescribing tree for low risk AF patients according to the CHA_2_DS_2_-VASC risk score. Hyperthy is hyperthyroidism.** Bmi is body mass index.
(TIFF)

Figure S2 **Warfarin prescribing tree for medium risk AF patients according to the CHA_2_DS_2_-VASC risk score.** ihd is ischemic heart disease. Bmi is body mass index. hf is heart failure.
(TIFF)

Figure S3 **Warfarin prescribing tree for high risk AF patients according to the CHA_2_DS_2_-VASC risk score.** hf is heart failure. mi is myocardial infarction. tia is transient ischemic attack. diab is diabetes. Ihd is ischemic heart disease.
(TIFF)
