# Supplementary figures and images for: Patterns of Warfarin Use in Subgroups of Patients with Atrial Fibrillation: A Cross-Sectional Analysis of 430 General Practices in the United Kingdom
Source: PLoS One. 2013 May 2;8(5):e61979. doi: 10.1371/journal.pone.0061979 (PMC3642100; doi:10.1371/journal.pone.0061979)

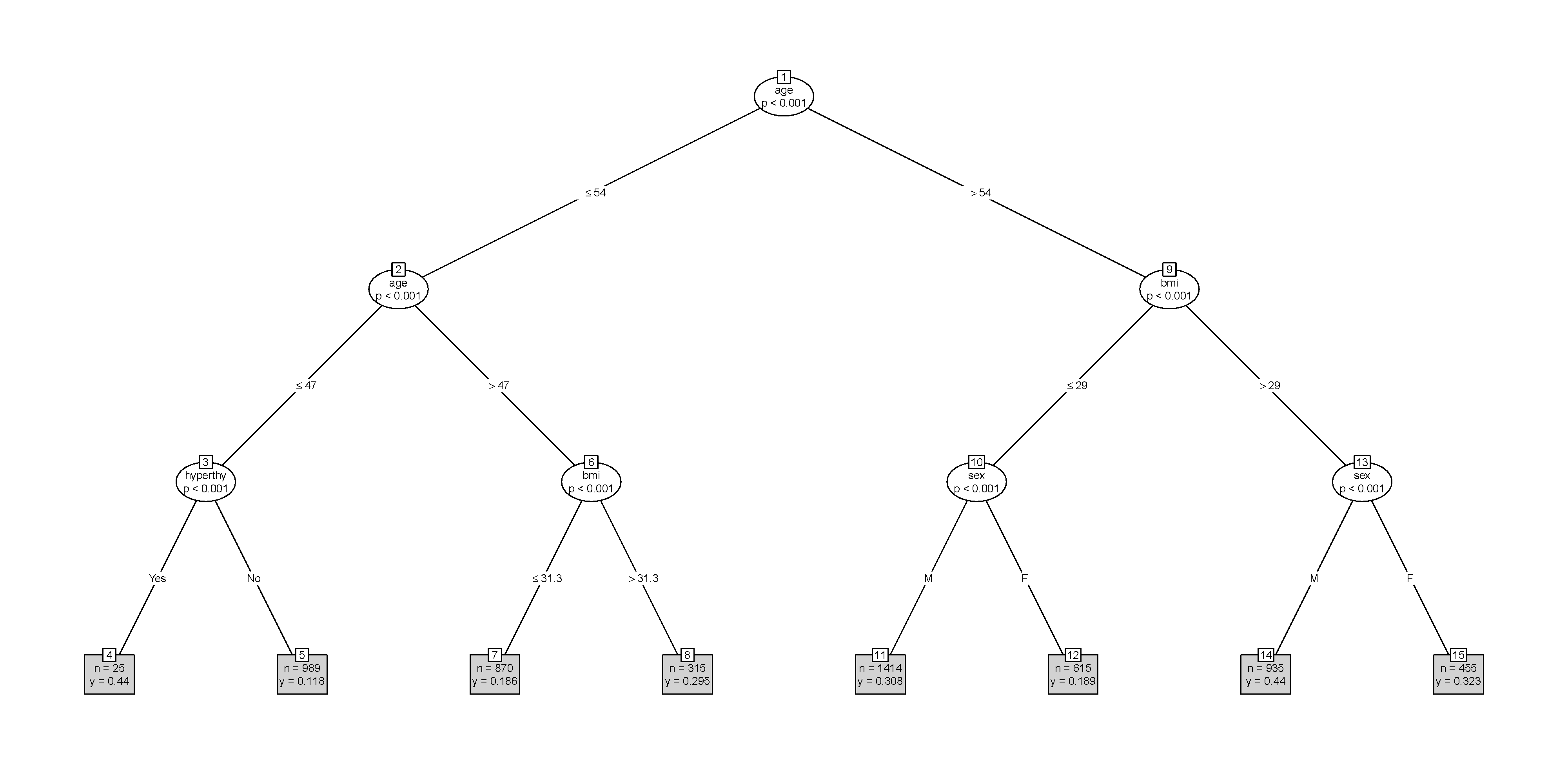

Supplement: Figure S1 — Warfarin prescribing tree for low risk AF patients according to the CHA2DS2-VASC risk score. Hyperthy is hyperthyroidism. Bmi is body mass index. (TIFF) [file pone.0061979.s001.tiff]

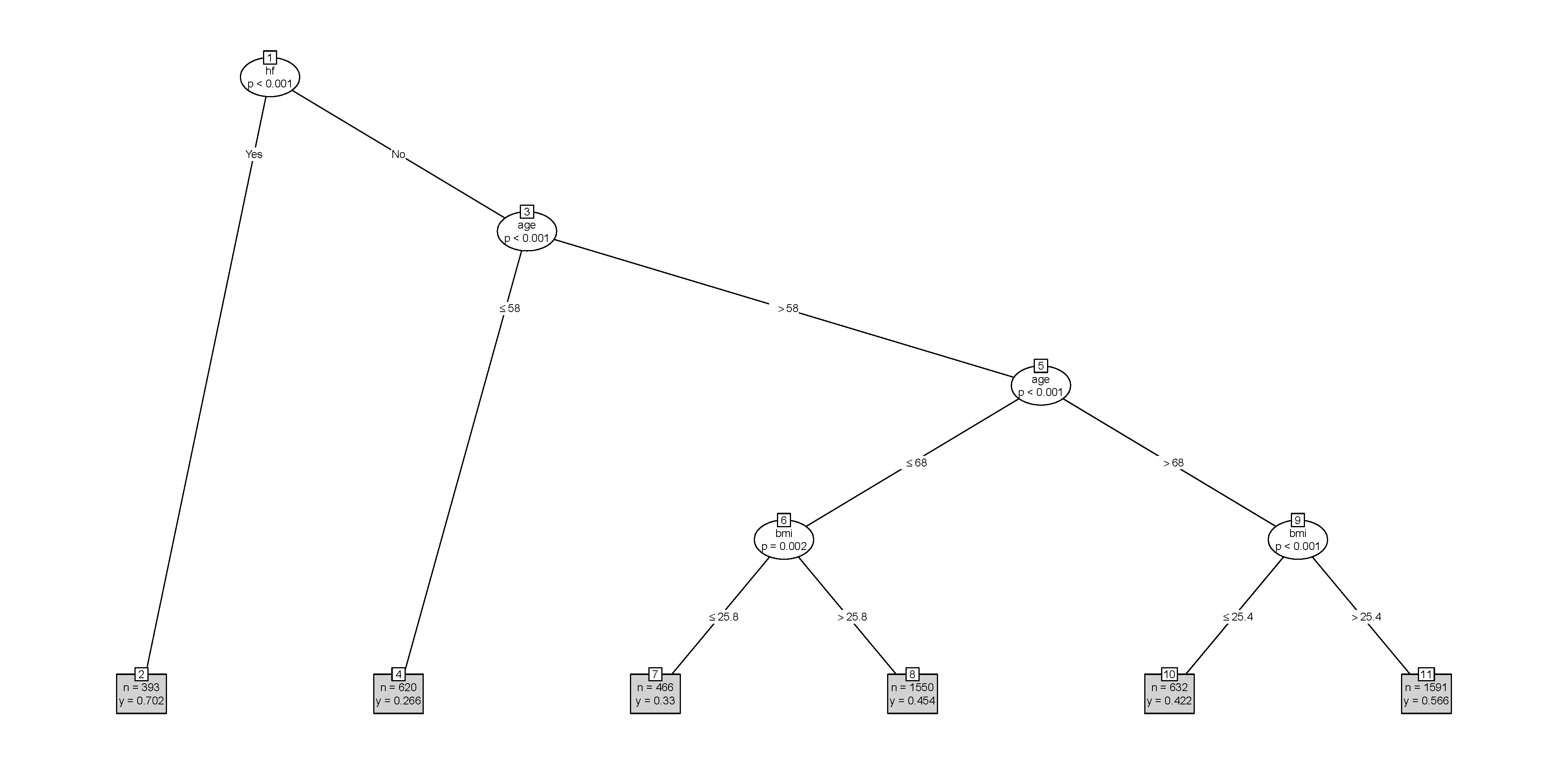

Supplement: Figure S2 — Warfarin prescribing tree for medium risk AF patients according to the CHA2DS2-VASC risk score. ihd is ischemic heart disease. Bmi is body mass index. hf is heart failure. (TIFF) [file pone.0061979.s002.tiff]

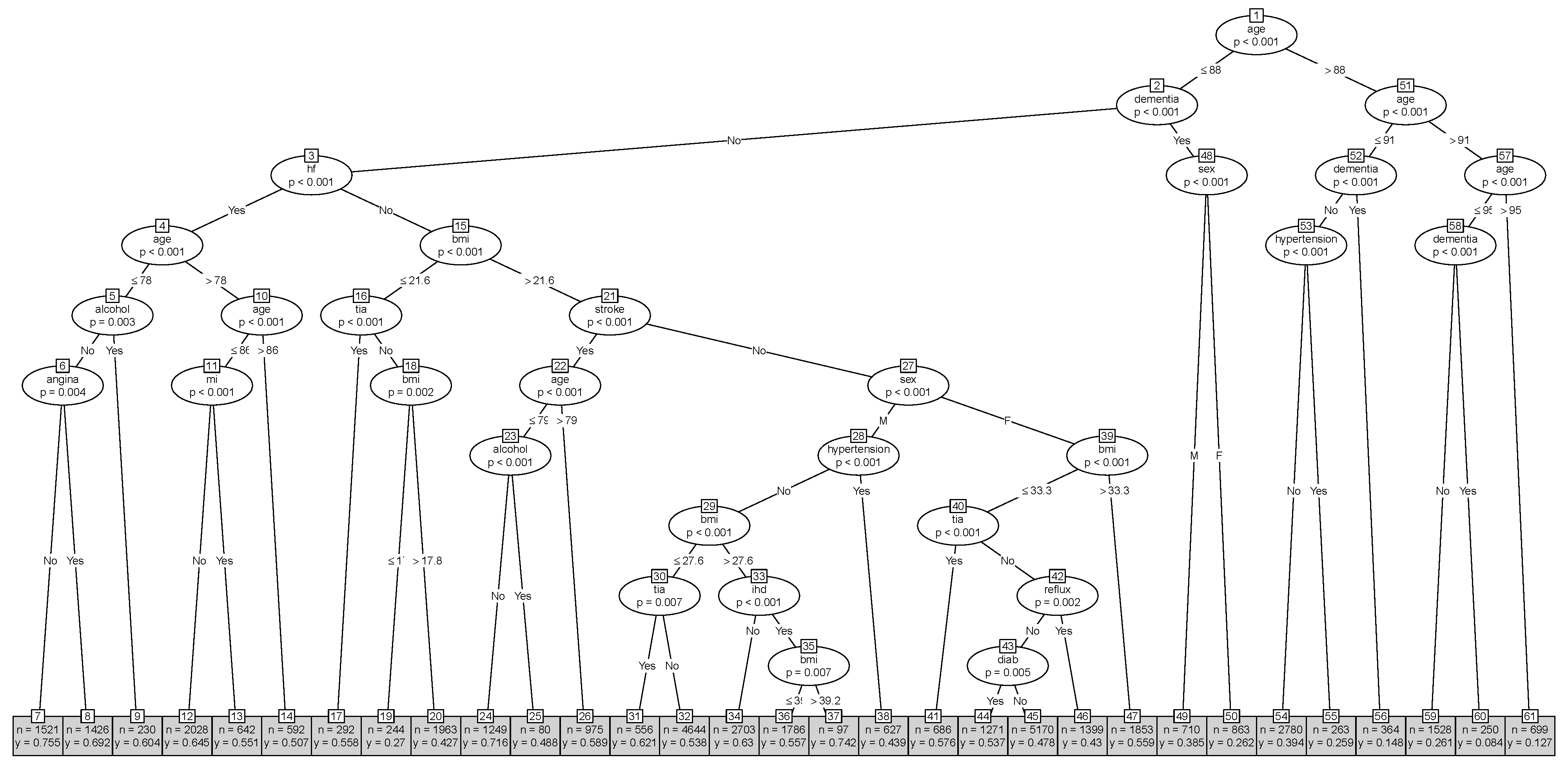

Supplement: Figure S3 — Warfarin prescribing tree for high risk AF patients according to the CHA2DS2-VASC risk score. hf is heart failure. mi is myocardial infarction. tia is transient ischemic attack. diab is diabetes. Ihd is ischemic heart disease. (TIFF) [file pone.0061979.s003.tiff]
